# *Yarrowia lipolytica* as an Oleaginous Platform for the Production of Value-Added Fatty Acid-Based Bioproducts

**DOI:** 10.3389/fmicb.2020.608662

**Published:** 2021-01-05

**Authors:** Huhu Liu, Yulan Song, Xiao Fan, Chong Wang, Xiangyang Lu, Yun Tian

**Affiliations:** College of Bioscience and Biotechnology, Hunan Agricultural University, Changsha, China

**Keywords:** *Yarrowia lipolytica*, cell factory, fatty acid metabolism, bioproducts, metabolic engineering

## Abstract

The microbial fermentation process has been used as an alternative pathway to the production of value-added natural products. Of the microorganisms, *Yarrowia lipolytica*, as an oleaginous platform, is able to produce fatty acid-derived biofuels and biochemicals. Nowadays, there are growing progresses on the production of value-added fatty acid-based bioproducts in *Y*. *lipolytica*. However, there are fewer reviews performing the metabolic engineering strategies and summarizing the current production of fatty acid-based bioproducts in *Y. lipolytica*. To this end, we briefly provide the fatty acid metabolism, including fatty acid biosynthesis, transportation, and degradation. Then, we introduce the various metabolic engineering strategies for increasing bioproduct accumulation in *Y. lipolytica*. Further, the advanced progress in the production of fatty acid-based bioproducts by *Y. lipolytica*, including nutraceuticals, biofuels, and biochemicals, is summarized. This review will provide attractive thoughts for researchers working in the field of *Y. lipolytica*.

## Introduction

With the growing crisis of oil energy, microbial production of biochemicals, as one potential alternative route, has received increasing attention (Levering et al., 2015; Ji and Huang, 2019; Ji and Ledesma-Amaro, 2020). Among these microorganisms, the oleaginous yeasts, such as *Rhodosporidium toruloides*, *Lipomyces starkeyi*, and *Yarrowia lipolytica*, are able to produce oleochemicals (Probst et al., 2016; McNeil and Stuart, 2018; Park et al., 2018b; Miller and Alper, 2019). *Y. lipolytica*, as Food and Drug Administration (FDA)-regarded Generally Recognized as Safe (GRAS) yeast with lipids over 20% of its biomass, performs many attractive characteristics and applications, including having mature genetic tools, secreting functional enzymes, and producing organic acids, lipids, and non-native chemicals (Xie, 2017; Darvishi et al., 2018; Larroude et al., 2018; Madzak, 2018; Ma et al., 2019). Currently, many researchers focus on the biotechnological application of *Y. lipolytica* (Xie et al., 2015; Markham et al., 2018; Robles-Rodriguez et al., 2018; Li et al., 2019). In particular, the different metabolic engineering strategies are applied in the lipid production for *Y. lipolytica* (Abdel-Mawgoud et al., 2018; Wang J. et al., 2020). In fact, *Y. lipolytica* is able to produce fatty acids in the form of lipids, either grown on hydrophilic or hydrophobic materials (Spagnuolo et al., 2018; Ma et al., 2020). Generally, these fatty acid-based bioproducts from *Y. lipolytica* are divided into three different types, based on the chain length, the terminal reductive state, and the modifications to the main chain of target product (Yan and Pfleger, 2020). With the development of metabolic engineering and synthetic biology, there are growing progresses on the production of value-added fatty acid-based bioproducts in *Y*. *lipolytica*. In the past 5 years, researchers have reviewed the production of fatty acid-derived products by *Y. lipolytica*, including fatty alkanes, fatty alcohols, and polyunsaturated fatty acids (PUFAs) (Ledesma-Amaro and Nicaud, 2016b; Ma et al., 2020). However, there is less review performing the metabolic engineering strategies for improving the production of fatty acid-based products and summarizing the current biosynthesis of fatty acid-based bioproducts in *Y. lipolytica*.

Herein, in this review, we describe a brief overview of the biochemistry metabolism of fatty acid in *Y. lipolytica*. Then, we focus on introducing the various metabolic strategies for increasing bioproduct accumulation, including constructing and engineering metabolic pathways, optimizing fermentation conditions, and engineering compartmentalization system. Moreover, we summarize the recent progress in the production of fatty acid-based bioproducts in *Y. lipolytica*, including nutraceuticals, biofuels, and biochemicals (Table 1). This article will provide attractive thoughts for researchers working in the field of *Y. lipolytica*.

**TABLE 1:** Summary of the production of fatty acid-based bioproducts from the *Y. lipolytica* platform.

**Type**	**Target**	**Strain**	**Genetic manipulation**	**Production level**	**References**
Nutraceuticals	DHA	*Y. lipolytica* Po1h:Af4	Expression of artificial *pfa*-BGC version C1_V2.	350 mg/L (after 300 h)	Gemperlein et al., 2019
	EPA	*Y. lipolytica* Y4305	Expression of C16 elongase gene, Δ12- desaturase gene, Δ9- elongase gene, Δ8- desaturase gene, Δ5- desaturase gene, Δ17- desaturase gene. Deletion of *PEX10* gene.	56.6% of total fatty acid	Xue et al., 2013
	EPA	*Y. lipolytica* Y4184	Deletion of Y*lsnf1*.	7.6% of the DCW	Seip et al., 2013
	EPA	*Y. lipolytica* Z7344	Expression of desaturases and elongases genes. Two-stage continuous fermentation.	48% of total lipids	Xie et al., 2017
	*Trans*-10, *cis*-12 CLA	*Y. lipolytica* Polh-1292*oPAI*-5	Expression of *PAI* gene.	5.9% of total fatty acid	Zhang B. X. et al., 2012
	*Trans*-10, *cis*-12 CLA	*Y. lipolytica* Polh-1292-spo*pai*-*d12*-16	Expression of *FADS12*, *d12* from *Mortierella alpine* and *opai* gene.	16% of DCW	Zhang B. X. et al., 2013
	CLA	*Y. lipolytica* JMY3479, CLIB 3039	Overexpression of *oPAI* and Δ12-desaturase from *Mortierella alpine*	302 mg/L	Imatoukene et al., 2017
	*Trans*-10, *cis*-12 CLA	*Y. lipolytica* WXYL037	Overexpression of inherent diacylglycerol transferase gene, Δ12-desaturase from *Mortierella alpina* and isomerase gene from *Propionibacterium acnes*.	132.6 mg/L	Wang et al., 2019
	GLA	*Y. lipolytica* pYLd6d12	Co-expression of fungal Δ6-desaturase and Δ12-desaturase genes	20% of GLA from endogenous LA and OA	Chuang et al., 2010
	GLA	*Y. lipolytica* Po1f-6-D	Expression of Δ6-desaturase gene from *Mortierella alpine*	71.6 mg/L	Sun et al., 2017
	ARA	*Y. lipolytica* YL 6-1	Expression of Δ6-desaturase, Δ6-elongase and Δ5- desaturase from *Mortierella alpine*.	0.4% of total lipids	Liu et al., 2017a
	ARA	*Y. lipolytica* YL 6-1	Transfer extracellular organic acids to the synthesis of intracellular ARA.	0.42% of total lipids	Liu et al., 2017b
	ARA	*Y. lipolytica* RH-4	Enzyme fusion of Δ9- elongase and Δ8- desaturase with the rigid linker (GGGGS)	118.1 mg/L	Liu H. H. et al., 2019
	RA	*Y. lipolytica* JMY2556	Expression of *CpFAH12* from *C. purpurea*. Overexpressing the native *LRO1*.	43% of total lipids	Beopoulos et al., 2014
	RA	*Y. lipolytica* CYLxR	Overexpression of *SCD1*, *DGA1*, *LIP2* and *CpFAH12*.	2.2 g/L	Guo et al., 2018
	Odd-chain FAs (C_17:1_)	*Y. lipolytica* CCY 29-26-36	Utilization propionate as substrate.	38% of total lipids	Kolouchová et al., 2015
	Odd-chain FAs (mainly C_15:0_, C_17:0_ and C_17:1_)	*Y. lipolytica* JMY3776	Overexpression of *ADH5*. Deletion of *ADH6*	0.57 g/L, 0.75 g/L (Fed-batch)	Park et al., 2018a
	Odd-chain FAs	*Y. lipolytica* JMY7412	Overexpression of the aspartate/α-ketobutyrate pathway	0.36 g/L	Park et al., 2020
Biofuels	Fatty alcohols (C_10_)	*Y. lipolytica* Δ*pex10*:*FATcpa*/*FAR*	Overexpression of *FAR* from *Arabidopsis thaliana* and *FAT* from *C*. *palustris*. Deletion of the major peroxisome assembly factor Pex10.	Over 500 mg/L	Rutter and Rao, 2016
	Fatty alcohols (C_16_)	*Y. lipolytica Tafar1*-5*copy*-Δ*dga1 fao1* strain	Expression of FAR gene from *Barn owl*.	636.89 mg/L (intracellular), 53.32 mg/L (extracellular)	Wang et al., 2016
	Fatty alcohols	*Y. lipolytica* Maqu2220-*Ec*fadD	Expression of fatty acyl-CoA reductase Maqu2220 from *Marinobacter aquaeolei* and *fadD* from *E*. *coli*. Compartmentalization	2.15 g/L (in a 3-L bioreactor)	Xu et al., 2016
	FAEE	*Y. lipolytica* AD strain	Expression of *Acinetobacter baylyi* ADP1 wax-ester synthase *Ab*AtfA. Overexpression of a peroxisomal/mitochondrial carnitine acyltransferase, perCat2. Mixtures of dextrose and canola oil. Compartmentalization	142.5 mg/L	Xu et al., 2016
	FAEE	*Y. lipolytica* GQY20	Expression of *WS* gene from *Marinobacter* sp. Deletion of *PEX10* gene.	1.18 g/L (containing 5 vol% ethanol)	Gao et al., 2018
	FAEE	*Y. lipolytica* YL6	Expression of *pdc* and *adhB* from *Z*. *mobilis* and maqu_0168 from *Marinobacter* sp. Deletion of *mfe1*, *gut2*, *pex10*. With vegetable cooking oils (VCOs).	82 mg/L	Ng et al., 2019
	FAEE	*Y. lipolytica* Po1g:pYLP1A1GAMh and S288C	Expression of *PDC1*, *ADH1*, *GAPDH* and *MhAtfA*. Co-culture.	4.8 mg/L	Yu et al., 2020
	C_19_ cyclopropanated fatty acids	*Y. lipolytica* ENGR-*HPH*:*ycoCFA*-*NAT*:*ycoCFA*	Expression of CFA synthase from *E*. *coli*.	3.03 g/L	Markham and Alper, 2018
	FFAs	*Y. lipolytica* JMY5743	Overexpression of *DGA2*, *TGL4*, *KlTGL3*. Deletion of *faa1*, *mfe1*.	10.4 g/L	Ledesma-Amaro et al., 2016
	FFAs	*Y. lipolytica* AD strain	Overexpression of hybrid hFAS-*Ec*TesA.	9.67 g/L (in a 3-L bioreactor)	Xu et al., 2016
	FFAs	*Y. lipolytica* Y-4311	Overexpression of *ACC1*. Deletion of *gpd1*, *gut2*, *pex10*.	2033.8 mg/L	Yuzbasheva et al., 2018
	Alkanes (C_5_)	*Y. lipolytica* PO1f-Δmfe1	Deletion of *mfe1*.	4.98 mg/L	Blazeck et al., 2013
	Alka(e)nes	*Y. lipolytica* AD strain	Expression of *Mm*CAR, *Bsu*Sfp and *Pm*ADO.	23.3 mg/L	Xu et al., 2016
	Alkenes (mainly C_15_ and C_17_)	*Y. lipolytica* S07004	Expression of *Cv*FAP (S121F) from *Chlorella variabilis*. Utilization half-light intensity.	58.7 mg/L (Fed-batch)	Bruder et al., 2019
Biochemicals	γ-decalactone	*Y. lipolytica* PO1d strain	Expression of acyl-CoA oxidase gene.	16.3 mg/g⋅h	Pagot et al., 1997
	γ-decalactone	*Y. lipolytica* Δ*pox2*Δ*pox3*	Deletion of *POX1* and *POX5* genes.	170 mg/L (2 L bioreactor)	Wache et al., 2001
	γ-decalactone	*Y. lipolytica* JMY185	Possession of multiple copies of *POX2* gene. Deletion of *POX3* and *POX5* genes.	150 mg/L	Waché et al., 2002
	γ-decalactone	*Y. lipolytica* W29	Increase O_2_ solubility	300 mg/L (2 L bioreactor)	Aguedo et al., 2005
	γ-decalactone	*Y. lipolytica* W29	Oxygen mass transfer in a biphasic medium.	141 mg/L (2 L bioreactor)	Gomes et al., 2007
	γ-decalactone	*Y. lipolytica* W29	Optimization operating conditions of substrate concentration, biotransformation start-up procedure and oxygen transfer.	87 mg/g⋅h	Gomes et al., 2010
	γ-decalactone	*Y. lipolytica* W29	Strategies of fed-batch culture.	73 mg/g (Intermittent fed-batch)	Gomes et al., 2012
	γ-decalactone	*Y. lipolytica* ATCC20460	Cell Immobilization.	1597 mg/L	Braga and Belo, 2013
	γ-decalactone	*Y. lipolytica* DSM 3286	Supply of oxygen	220 mg/L (Fed-batch)	Moradi et al., 2013
	γ-decalactone	*Y. lipolytica* G3-2.21	Genome shuffling of the haploid cells and the parent strains CGMCC 2.1405.	3.75 g/L	Zhao et al., 2014
	γ-decalactone	*Y. lipolytica* W29	The direct influence of oxygen transfer rate.	215 g/L (Fed-batch)	Braga and Belo, 2014
	γ-decalactone	*Y. lipolytica* w-YLG	Cell immobilization in attapulgite along with the use of ionic liquid as a cosolvent.	8.05 g/L (Fed-batch)	Zhao et al., 2015
	γ-decalactone	*Y. lipolytica* CCMA 0242	Optimization of cultivation conditions.	0.128 g/L	Pereira de Andrade et al., 2017
	γ-decalactone	*Y. lipolytica* CCMA 0357	Optimization of cultivation conditions.	3.5 g/L	Soares et al., 2017
	γ-decalactone	*Y. lipolytica* CGMCC 2.2087	Cell immobilization with BC-ALG carriers.	8.37 g/L	Zhang et al., 2020
	δ-decalactone	*Y. lipolytica* KCTC 17170	Expression of linoleate 13-hydratase from *L*. *acidophilus*.	16.3 mg/(L⋅h)	Kang et al., 2016
	HFAs (ω-HDDA)	*Y. lipolytica* H222ΔPΔAΔF	Deletion of *POX1-6*, all relevant *ADH* genes and *FAO1*.	7.9 g/L	Gatter et al., 2014
	DCAs (C_12_)	H222ΔP	Deletion of *POX1-6*.	11 g/L	Gatter et al., 2014
	DCAs (C_12_)	*Y. lipolytica* iYLI647	*In silico* model-based metabolic engineering.	ND	Mishra et al., 2018
	DCAs (C_12_)	*Y. lipolytica* MTLY 37	Deletion of *pox2*, *pox3*, *pox4*, *pox5*.	20 mg/mL	Smit et al., 2005
	Hexanal	*Y. lipolytica* PO1d-HPL	Expression of *HPL* gene.	350 mg/L (Reaction medium)	Bourel et al., 2004
	Hexanal	*Y. lipolytica* JMY 861	Expression the hydroperoxide lyase (*HPL*) gene from green bell pepper fruit. Under oxido-reducing conditions.	600 mg/L	Santiago-Gómez et al., 2009
	Hexanal	*Y. lipolytica* JMY 861	Overexpression of ADH from *S*. *cerevisiae*.	Increased by 84.1%	Aziz et al., 2016
	CFA (C_17_ and C_19_)	*Y. lipolytica* JMY 6068	Expression of *CFAs* from *E*. *coli*.	2319 mg/L	Czerwiec et al., 2019

*ND, not determine.*

## Biochemistry of Fatty Acid Metabolism

**FIGURE 1:**
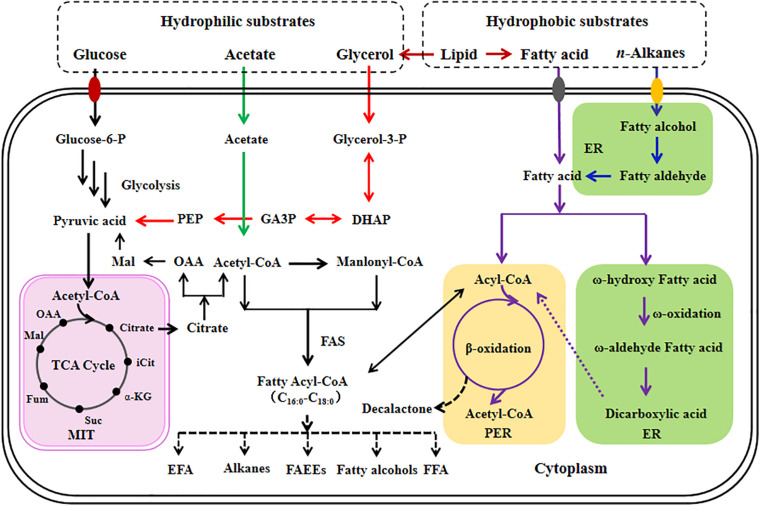
Overview of fatty acid metabolism for the production of its based chemicals in *Y. lipolytica*. Different colored arrows are used to represent different metabolic pathways; black, *de novo* fatty acid metabolic pathway; green, acetate metabolic pathway; red, glycerol metabolic pathway; dark red, heterologous lipid metabolic pathway; purple, *ex novo* fatty acid metabolic pathway; blue, heterologous alkane metabolic pathway. Pathway localization with respect to specific subcellular organelles are also depicted. ER, endoplasmic reticulum; PER, peroxisome; MIT, mitochondria; TCA cycle, tricarboxylic acid cycle; DHAP, dihydroxyacetone phosphate; GA3P, glycerol-3-phosphate; iCit, isocitrate; α-KG, α-ketoglutarate; Suc, succinate; Fum, fumarate; Mal, malate; OAA, oxaloacetate; FAS, fatty acid synthase; EFA, essential fatty acid; FFA, free fatty acid; FAEEs, fatty acid ethyl esters.

Currently, some articles have summarized the fatty acid metabolism of *Y. lipolytica* (Fickers et al., 2005; Abghari and Chen, 2014; Ledesma-Amaro and Nicaud, 2016a; Lazar et al., 2018). Previously, we reviewed in detail the characteristics of *Y. lipolytica* grown on various carbon substrates (Liu et al., 2015). Herein, the metabolism of fatty acid for producing its derived chemicals in *Y. lipolytica* is shown in Figure 1.

### Fatty Acid Biosynthesis

With the development of metabolic engineering, it enables *Y. lipolytica* to utilize a wide range of carbon sources (Liu et al., 2015; Ledesma-Amaro and Nicaud, 2016b). Using hydrophilic substrates (such as glucose and glycerol) as carbon source, fatty acid is synthesized by *de novo* pathway in *Y. lipolytica*. With glucose as sole carbon source, it is converted into pyruvate *via* the glycolytic pathway in the cytosol. Then, pyruvate is transported to mitochondria and transformed into acetyl-CoA. Acetyl-CoA, a key precursor involved in fatty acid biosynthesis, can be produced by different metabolic routes, including citrate degradation catalyzed by ATP citrate lyase (ACL), fatty acid degradation from β-oxidation pathway, acetate transformation by acetyl-CoA synthetase (ACS, YALI0F05962p), and pyruvate transformation by pyruvate dehydrogenase complex. Under nitrogen-limited conditions, citrate is secreted into cytosol from mitochondria in *Y. lipolytica* and acetyl-CoA is produced by ACL catalysis. In *Y. lipolytica*, ACL is encoded by *ACL1* (YALI0E34793p) and *ACL2* (YALI0D24431p). Further, acetyl-CoA is transformed into malonyl-CoA by acetyl-CoA carboxylase (ACC, YALI0C11407p). Generally, acetyl-CoA and malonyl-CoA are used as substrates for fatty acid biosynthesis by fatty acid synthetases (FAS, YALI0B15059p, and YALI0B19382p) in *Y. lipolytica*. Naturally, *Y. lipolytica* can only produce C_16_ and C_18_ fatty acids (Beopoulos et al., 2009). Notably, the inherent long-chain PUFAs, including oleic acid (OA, C_18__:__1_) or linoleic acid (LA, C_18__:__2_), are synthesized by desaturase located in endoplasmic reticulum (ER).

Using hydrophobic materials (such as fats) as substrate, fatty acids are synthesized by *ex novo* pathway in *Y. lipolytica*. Generally, the extracellular fatty acids from the metabolism of hydrophobic materials are directly transported to cytosol in *Y. lipolytica*. Then, fatty acids are converted into derived chemicals by the corresponding oxidation process. Additionally, using alkane from oil refinery as carbon source, fatty acids are synthesized by the enzyme catalytic system located in ER, including cytochrome P450 reductase (EC 1.6.2.4), fatty alcohol oxidase (EC 1.1.3.20), and fatty aldehyde dehydrogenase (EC 1.2.1.3).

NADPH is an important reducing power involved in fatty acid biosynthesis in *Y. lipolytica*. Generally, there are two identified routes for providing NADPH pool in *Y. lipolytica* (Qiao et al., 2017). One route is from decarboxylation reaction catalyzed by malic enzyme (EC 1.1.1.40) that occurred in cytosol; the other metabolic route is from the pentose phosphate pathway in *Y. lipolytica*. Previously, it was reported that overexpression of malic enzyme has little impact on lipid accumulation in *Y. lipolytica* (Beopoulos et al., 2011; Zhang H. et al., 2013). Wasylenko et al. (2015) reported that the oxidative pentose phosphate pathway, harboring glucose 6-phosphate dehydrogenase (EC 1.1.1.49) and 6-phosphogluconolactonase (EC 3.1.1.31), is the primary source of lipogenic NADPH in *Y. lipolytica*.

### Fatty Acid Transportation

To date, the mechanism of fatty acid transportation is unclear in *Y. lipolytica*. Generally, shorter carbon-chain fatty acids, such as C_8__:__0_ and C_10__:__0_, are toxic for *Y. lipolytica*. Using primrose oil containing C_18_ fatty acids as substrate, *Y. lipolytica* performs a higher assimilation rate for unsaturated fatty acids (C_18__:__3_, C_18__:__2_, and C_18__:__1_) than that for saturated fatty acid (C_18__:__0_) (Aggelis et al., 1997). In this research, it was deduced that the fatty acids with different saturated levels are assimilated and transported *via* a selective uptake mechanism in *Y. lipolytica*. Recently, Dulermo et al. (2015) proposed a model of fatty acid transportation with chain length preferences in *Y. lipolytica*. According to this model, the extracellular fatty acids are transported into *Y. lipolytica via* unidentified transporters. Then, the internal fatty acids are activated to acyl-CoA by YlFaa1p (YALI0D17864p) or transported into peroxisome by unknown transporters. Notably, the activated fatty acids can be sorted in the form of triacylglycerols or enter peroxisome *via* transporters YlPxa1p (YALI0A06655p) and YlPxa2p (YALI0D04246p). Importantly, fatty acids from lipid remobilization can enter the peroxisome *via* transporter YlFat1p (YALI0E16016p).

In particular, the intracellular medium-chain fatty acids (C_12_–C_14_) are converted into fatty acyl-CoAs by fatty acyl-CoA synthetase II in the peroxisome for further degradation, whereas long-chain fatty acids (C_16_–C_18_) are converted into fatty acyl-CoA by fatty acyl-CoA synthetase I in the cytosol (Dulermo et al., 2015). Then, long-chain fatty acyl-CoA is either transported into peroxisome from cytosol or used as substrate for triacylglyceride biosynthesis in *Y. lipolytica*.

### Fatty Acid Degradation

Generally, fatty acids, either from intracellular triacylglyceride hydrolysis or from extracellular fatty acid transportation, can be transformed into fatty acid-based chemicals by oxidation in *Y. lipolytica*. Notably, the intracellular fatty acids are mainly degraded by peroxisomal β-oxidation or ω-oxidation pathway. In fact, the intracellular fatty acids from lipid remobilization are mainly converted into acetyl-CoA, *via* peroxisomal β-oxidation pathway. In particular, each cycle of β-oxidation consists of a four-step enzyme catalyzed reaction in *Y. lipolytica*. The first step is catalyzed by acyl-CoA oxidases (EC 1.3.3.6), the second step and third steps are catalyzed by multifunctional enzyme, and the last step is catalyzed by 3-ketoacyl-CoA thiolase (EC 2.3.1.16). In addition, the intracellular fatty acids can be degraded into derived chemicals by ω-oxidation pathway that occurred in ER. The fatty acids are firstly converted into ω-hydroxyl-fatty acids by cytochrome P450-containing fatty acid ω-hydroxylase. Then, ω-hydroxyl-fatty acids are converted into ω-aldo-fatty acids by fatty alcohol dehydrogenase or fatty alcohol oxidase, and ω-aldo-fatty acids are converted into long-chain diacids by fatty aldehyde dehydrogenase. In particular, the β-oxidation pathway can be engineered to synthesize β-hydroxy fatty acid (HFA) and lactones, whereas the ω-oxidation pathway can be engineered to produce ω-HFA and α, ω-dicarboxylic acids (DCAs) in *Y*. *lipolytica*.

## Engineering Strategies to Increase Oleochemical Production

Nowadays, different metabolic strategies have been used to *de novo* produce the novel fatty acid-based bioproducts and accumulate the production of these derived biochemicals in *Y*. *lipolytica* (Table 2).

**TABLE 2:** Engineering strategies to improve fatty acid-based bioproducts accumulation in *Y*. *lipolytica.*

**Engineering strategies**	**Bioproducts**	**Strategy details**	**References**
Constructing and engineering metabolic pathways	EPA	Constructing synthetic pathways	Xue et al., 2013
	Lipids	Improving acetyl-CoA supplement	Xu et al., 2016
	Lipids	Increasing NADPH availability	Qiao et al., 2017
	*Trans*-10, *cis*-12 CLA	Overexpressing the endogenous enzymes	Wang et al., 2019
	Fatty alcohols	Eliminating downstream degradation	Rutter and Rao, 2016
Optimizing fermentation conditions	γ-decalactone	Improving oxygen transfer	Moradi et al., 2013
	GLA	A temperature-shift strategy of cultivation	Sun et al., 2017
	CLA	Changing the medium components	Wang et al., 2019
	EPA	Two-stage continuous fermentation	Xie et al., 2017
Engineering compartmentalization system	FAEE	Endoplasmic reticulum or peroxisome localization	Xu et al., 2016
	Alkane	Endoplasmic reticulum or peroxisome localization	
	Fatty alcohol	Peroxisome localization	
	γ-decalactone	Cell immobilization	Zhang et al., 2020

### Constructing and Engineering Metabolic Pathways

Researchers have focused on constructing and optimizing metabolic pathways to achieve efficient fatty acid and its derivatives biosynthesis in *Y*. *lipolytica*, using various metabolic engineering strategies, including constructing heterologous synthetic pathways, overexpressing endogenous enzymes. Naturally, *Y*. *lipolytica* can produce linoleic acid as the precursor of ω-3/6 fatty acids (Liu et al., 2017a). Generally, the novel linoleic acid-derived nutraceuticals, such as arachidonic acid (ARA, C_20__:__4_) and eicosapentaenoic acid (EPA, C_20__:__5_), can be *de novo* synthesized *via* constructing the synthetic pathway in *Y*. *lipolytica*. For example, to *de novo* produce EPA in *Y*. *lipolytica*, the selected and optimized multiple copies of different chimeric genes from different microorganisms were integrated into yeast genome (Δ9-elongase, Δ8-desaturase, and Δ5-desaturase from *E*. *gracilis*, C16/18-elongase from *M*. *alpina*, Δ12-desaturase gene from *F*. *moniliforme*, Δ17-desaturase from *P*. *aphanidermatum*, and *CPT*), which led to the first engineered commercial strain Y4305 under strong promoters, containing 30 copies of nine different genes, which can produce EPA at 56.6% of the total fatty acids (TFA), without γ-linolenic acid (GLA, C_18__:__3_) accumulation (Xue et al., 2013).

Through overexpressing and eliminating the endogenous enzymes involved in the lipid degradation, the accumulation of fatty acid and its derivatives has been greatly enhanced in *Y*. *lipolytica* (Dulermo and Nicaud, 2011). Generally, the availability of precursors, including acetyl-CoA and NADPH, limits the lipid biosynthesis. Previously, by harnessing the carnitine shuttle mechanism, the lipid titer was enhanced 1.75-fold *via* increasing acetyl-CoA supplement (Xu et al., 2016). Qiao et al. (2017) performed a specific strategy of converting NADH to NADPH in 13 engineered strains of *Y*. *lipolytica* for improving lipid synthesis. Recently, Wang et al. (2019) showed that the increased conjugated linoleic acid (CLA, C_18__:__2_) accumulation is reached by overexpressing the endogenous diacylglycerol transferase gene. Additionally, in order to block the lipid degradation in *Y*. *lipolytica*, Rutter and Rao (2016) showed that the peroxisome assembly factor Pex10 is the major enzyme involved in the peroxisomal β-oxidation or ω-oxidation pathway.

### Optimizing Fermentation Conditions

The optimization of fermentation process, based on the microbial physiology, plays a key role in achieving the high titer, yield, and productivity of value-added products. Naturally, pH, temperature, and medium components are the common optimized approaches during the fermentation process of *Y*. *lipolytica*. Previously, the temperature-shift strategy of cultivation was successfully exhibited to increase GLA accumulation in *Y*. *lipolytica* (Sun et al., 2017). Recently, the production of CLA was increased by changing carbon and nitrogen source, carbon-t- nitrogen mass ratio, and CaCl_2_ concentrations (Wang et al., 2019). In addition, the fed-batch fermentation approach has been used to increase the production of drop-in biochemicals (Park et al., 2018a; Bruder et al., 2019). Compared with the continuous fermentation processes, the batch and fed-batch processes perform lower volumetric productivities (Li et al., 2011). In fact, the productivities utilizing continuous fermentation processes were improved, typically at the cost of product concentration, conversion yield, or both (Ethier et al., 2011). Previously, the novel two-stage continuous process for EPA accumulation in *Y*. *lipolytica* was developed (Xie et al., 2017). In this research, compared with the single-stage continuous and fed-batch fermentation, the novel continuous process, equipped with a small growth tank (Stage 1) and a large production tank (Stage 2), successfully improved the volumetric lipid productivities by 80%.

Generally, *Y*. *lipolytica* requires a high oxygen supply in the large-scale bioprocess. Previously, researchers have showed that the heterologous expression of gene encoding the bacterial hemoglobin from *Vitreoscilla stercoraria* (VHb) can improve the oxygen utilization efficiency and further increase the productivity (Suen et al., 2014; Zhang et al., 2017). Recently, Mirończuk et al. (2019) performed that the improved erythritol synthesis is obtained in *Y*. *lipolytica*, by overexpressing the codon-optimized bacterial hemoglobin (VHb). Through improving oxygen transfer rate using higher agitation rates or pure oxygen for aeration, the production of γ-decalactone was successfully enhanced (Moradi et al., 2013).

### Engineering Compartmentalization System

Naturally, each subcellular compartment in *Y*. *lipolytica* provides a unique microenvironment, including enzyme, precursor, and cofactor composition. Due to the distinct organelle characteristics, the separation of organelles in the cytosol performs the potential to eliminate metabolic crosstalk and enhance compartmentalized pathway efficiency (Hammer and Avalos, 2017). Previously, Xu et al. (2016) reported that the titer of drop-in product performs a 10–15-fold improvement, by targeting the fatty acid ethyl ester (FAEE) pathway to either ER or peroxisome of *Y*. *lipolytica*. Compared to free cell systems, the immobilized cells could tolerate unsuitable conditions (Li et al., 2009; Macario et al., 2009). For example, using cell immobilization systems with bacterial cellulose-alginate (BC-ALG) carriers, γ-decalactone production was successfully reached with 8.37 g/L in the repeated experiments in *Y. lipolytica*, an approximately 3.7-fold improvement over with an ALG carrier alone (Zhang et al., 2020).

Modular co-culture metabolic engineering combines the strains carrying each pathway module in the engineered strains to form a synthetic complex, which can accommodate different modules expressing functional genes in different hosts to produce drop-in bioproducts (Jawed et al., 2019). Recently, by coculturing and engineered *Y*. *lipolytica* and *S*. *cerevisiae* strain, a synthetic microbial consortium was constructed to increase the titer of FAEE. In this research, the titer of FAEE biodiesel at 4.8 mg/L was reached by the synthetic microbial consortium under the optimum coculture conditions (Yu et al., 2020).

## Production of Fatty Acid-Based Bioproducts

### Nutraceuticals

Due to the potential applications of microbial lipids in the field of food supplements, the microbial production of PUFAs is becoming an industrial reality (Bellou et al., 2016). Of these oleaginous yeasts, *Y. lipolytica* can synthesize OA and LA.

Omega-3 PUFAs with special function, particularly α-linolenic acid (ALA, C_18__:__3_), EPA, and docosahexaenoic acid (DHA, C_22__:__6_), are gaining importance. Previously, using inherent LA as carbon substrate, Xue et al. (2013) constructed an engineered *Y. lipolytica* strain Y4305 capable of *de novo* producing EPA at 56.6% of TFA, by the combined metabolic engineering strategies. With *Y. lipolytica* as a host, the highest titer of ALA at 1.4 g/L was produced in the engineered strain containing a bifunctional Δ12–Δ15 desaturase from *Rhodosporidium kratochvilovae*, under the optimized fermentation conditions (Cordova and Alper, 2018). Recently, an artificial PUFA biosynthetic gene clusters, encoding DPA/DHA-type PUFA synthases, was expressed in *Y. lipolytica*. In this research, under the optimized fermentation process, the DHA level over 350 mg/L was reached (Gemperlein et al., 2019).

Omega-6 PUFAs, including conjugated CLA, GLA, and ARA, are a major family of PUFAs with diverse bioactivities (Xu and Qian, 2014). In 2017, the combined elimination of β-oxidation pathway and overexpression of Δ12-desaturase was conducted in *Y. lipolytica*, which leads to CLA production at 302 mg/L (Imatoukene et al., 2017). Recently, Wang et al. (2019) showed that the maximum content of *trans*-10, *cis*-12 CLA at 132.6 mg/L is reached by the engineered *Y. lipolytica* under the optimized fermentation conditions, by the overexpression of inherent diacylglycerol transferase from *Y. lipolytica*, Δ12 desaturase from *Mortierella alpina*, and *Propionibacterium acnes* isomerase. With LA as substrate, the GLA biosynthetic pathway was constructed in *Y. lipolytica* harboring Δ6-desaturase from *M. alpina*. Under the optimized fermentation process, the titer of GLA at 71.6 mg/L was achieved (Sun et al., 2017).

Arachidonic acid (ARA, C_20__:__4_) is also an essential ω-6 PUFA with special functions. Previously, we developed the *in vivo* one-step pathway assembly and integration method enabling *Y. lipolytica* to produce ARA (Liu et al., 2017a). Additionally, we showed that the ARA biosynthetic pathway is able to redirect the carbon flux toward intracellular fatty acid accumulation at the expense of extracellular organic acid secretion in the engineered *Y. lipolytica* strain (Liu et al., 2017b). Recently, using Δ9 elongase pathway engineering and fusion enzyme strategy, the ARA titer at 118.1 mg/L was achieved in the engineered *Y. lipolytica* (Liu H. H. et al., 2019).

Ricinoleic acid (RA, C_18__:__1_) and its derivatives perform oleochemical applications, due to the special characteristics. Meesapyodsuk and Qiu (2008) first identified an oleic acid-like hydroxylase (*CpFAH12*) from *Claviceps purpurea*. Previously, with LA as substrate, an engineered *Schizosaccharomyces pombe* strain capable of producing RA, harboring heterologous *CpFAH12* from *C*. *purpurea*, was constructed (Holic et al., 2012). Using *Y. lipolytica* as a host, Beopoulos et al. (2014) reported that RA accumulation at 42% of total lipids is achieved, by overexpressing *C*. *purpurea* Δ12-hydroxylase and native *Y. lipolytica* Lro1p acyltransferase. Recently, by the combined overexpression of *SCD1* gene encoding stearoyl-CoA desaturase, *DGA1* gene encoding acyl-CoA:diacylglycerol acyltransferase, *LIP2* gene encoding lipase, and *CpFAH12* gene encoding hydroxylase, the production level of RA at 2.2 g/L was obtained by the engineered *Y. lipolytica* using cellulose as substrate (Gao et al., 2018).

Odd-chain fatty acids with special biochemical and biological activities are receiving growing attention on potential applications (Řezanka and Sigler, 2009). Previously, Kolouchová et al. (2015) performed that *Y. lipolytica* is capable of producing heptadecenoic acid (C_17__:__1_) using propionate as substrate. Recently, the deletion of the *PHD1* gene and optimization of the fermentation process were applied to produce odd-chain fatty acids (mainly C_15__:__0_, C_17__:__0_, and C_17__:__1_) by *Y. lipolytica* grown on propionate (Park et al., 2018a). Additionally, Park et al. (2020) constructed an engineered *Y. lipolytica* capable of *de novo* producing odd-chain fatty acids, using glucose as sole substrate without any propionate supplementation.

### Biofuels

The microbial production of fatty alcohols is becoming an alternative method to meet the increasing demand. Presently, various microorganisms, such as *Escherichia coli* and *Saccharomyces cerevisiae*, have been engineered for fatty alcohol production (Zhang et al., 2011; Zhou et al., 2016). Using *Y. lipolytica* as a host, Wang et al. (2016) constructed a novel fatty alcohol-producing workhorse, harboring *Tafar1* gene coding fatty acyl-CoA reductase. Under the optimized tri-module condition, the intracellular hexadecanol at 636.89 mg/L and extracellular hexadecanol at 53.32 mg/L was produced, respectively. Meanwhile, through the overexpression of fatty acyl-ACP-thioesterases and fatty acyl-CoA reductase, and deletion of the major peroxisome assembly factor Pex10, the medium-chain alcohol, especially 1-decanol over 500 mg/L, was produced in the engineered *Y. lipolytica* (Rutter and Rao, 2016).

Researchers have performed that FAEEs or fatty acid methyl esters (FAMEs) can be produced *via* the microbial fermentation, using *E. coli* and *S. cerevisiae* (Steen et al., 2010; Nawabi et al., 2011; Yu et al., 2012). Fortunately, Xu et al. (2016) reported that the highest titer of FAEEs at 142.5 mg/L is produced in the engineered *Y. lipolytica*, using the compartmentalized metabolic engineering. Recently, an engineered *Y. lipolytica* strain, harboring the heterogenous pyruvate decarboxylase (*pdc*), alcohol dehydrogenase II (*adhB*) from *Zymomonas mobilis*, and wax ester synthases from *Marinobacter* sp., was constructed for producing FAEE. In this research, the titer of FAEE up to 82 mg/L was achieved by the supplementation of vegetable cooking oil (Ng et al., 2019). Meanwhile, Yu et al. (2020) developed the synthetic co-culture system comprising the engineered *S. cerevisiae* and *Y. lipolytica* strain, which was able to produce FAEE at 4.8 mg/L. To overcome the limitation of oxidative stability in the traditional FAMEs, Markham and Alper (2018) first performed the production of C19 cyclopropanated fatty acids in the engineered *Y. lipolytica* strain, harboring the heterologous cyclopropane fatty acid synthase from *E. coli*. In this research, the titer of C19 cyclopropanated fatty acids over 3.0 mg/L was produced under the bioreactor fermentation.

Free fatty acids (FFAs) are special oleochemicals with wide applications in the field of agricultural chemicals, soaps, and surfactants. Previously, Zhou et al. (2016) engineered *S. cerevisiae* capable of producing FFAs. Using *Y. lipolytica* as a workhorse, FFAs up to 9.67 g/L were produced by the engineered strain under the bioreactor scale with pH control (Xu et al., 2016). With the mixture of glucose and glycerol as carbon source, Yuzbasheva et al. (2018) showed that the engineered *Y. lipolytica* Y-4311 strain can produce FFAs (2033.8 mg/L) by the addition of dodecane.

Alka(e)nes are the major components of gasoline, diesel, and jet fuel. Presently, many studies have explored that the microbial production of alkanes is a conceivable method (Choi and Lee, 2013; Zhou et al., 2016). Using *Y. lipolytica* as a host expressing soybean lipoxygenase enzyme, Blazeck et al. (2013) first developed a microbial platform capable of producing pentane. In particular, in this research, using LA as substrate, the high titer of pentane at 4.98 g/L was produced. Recently, Bruder et al. (2019) revealed that the engineered *Y. lipolytica* is able to produce odd-numbered alkanes and alkenes (mainly C15 and C17), by the expression of light-driven oxidase. Interestingly, using the lighting bioreactors, the titer of alkenes at 58.7 mg/L was first reached in this research.

### Biochemicals

γ-decalactone, a well-known aroma compound, is mainly synthesized *via* β-oxidation. Previously, we have summarized in detail the γ-decalactone production by *Y. lipolytica* (Liu et al., 2015). Recently, using the immobilized culture technology, the maximum production of γ-decalactone reached 8.37 g/L by *Y*. *lipolytica* strain on bacterial cellulose-alginate carriers (Zhang et al., 2020). Additionally, using a one-pot biotransformation process containing whole *Y. lipolytica* cells, the highest production of δ-decalactone at 58.7 mg/L was first performed (Kang et al., 2016).

HFAs, as valuable building blocks, can be synthesized by the biotransformation of fatty acids *via* the terminal carbon oxygenation (Seo et al., 2015). To date, the microbial production of ω-HFAs by the engineered *E. coli* has received specific progress (Kim and Park, 2019). Using *Y. lipolytica* as a promising workhorse, an engineered strain capable of synthesizing ω-hydroxy dodecanoic acid was constructed, through the deletion of acyl-CoA oxidase-coding genes (*POX 1–6*), fatty alcohol oxidase gene (*FAO1*), and alcohol dehydrogenase genes (*ADH 1–8*) (Gatter et al., 2014). Recently, Rigouin et al. (2019) showed that the engineered *Y. lipolytica* is able to produce polyhydroxyalkanoates composed of 3-HFAs, using methyl myristate as precursor.

DCAs are also important intermediates in the industrial field. At present, the microbial production of DCAs, as an alternative method, are gaining interests (Huf et al., 2011; Ledesma-Amaro and Nicaud, 2016b; Werner and Zibek, 2017). *Y*. *lipolytica* can produce DCAs *via* alkane degradation (Nicaud et al., 2006). Previously, researchers have shown that the engineered *Y. lipolytica* can produce dioic acids (Smit et al., 2005; Nicaud et al., 2006). In particular, Gatter et al. (2014) showed that the overexpression of *FAO1* leads to an improved production of dodecane dioic acid at 11 g/L. Recently, using the *in silico* model-based metabolic engineering strategies, the metabolic flux toward DCAs production was obviously increased in *Y. lipolytica* (Mishra et al., 2018).

Hexanal, one of C-6 aldehydes with green odor, can be synthesized *via* the degradation from LA using lipoxygenase and hydroperoxide lyase. Previously, using *Y. lipolytica* as a host, Bourel et al. (2004) showed that hexanal is produced by expressing of fatty acid hydroperoxide lyase. Further, Santiago-Gómez et al. (2009) reported the effect of oxido-reduction environment on hexanal production. Interestingly, in this research, under the optimized conditions, the highest titer of hexanal at 600 mg/L was produced by the engineered *Y. lipolytica.*

In addition, cyclopropane fatty acids (CFAs), as good unusual fatty acid candidates, were produced by the engineered *Y*. *lipolytica* (Czerwiec et al., 2019). In this research, by expressing genes from various organisms and optimizing the expression level of CFAs synthase and fed-batch fermentation, it was shown that CFAs at 2319 mg/L (mainly C17:0 and C19:0 cyclopropanated form) are finally synthesized in the strain JMY 6068. Compared with *E. coli* and *S. cerevisiae*, the fatty acid derivatives produced by *Y*. *lipolytica* are more abundant (Table 3).

**TABLE 3:** Comparison of the productivity of fatty acid-derived biofuels between *E*. *coli*, *S*. *cerevisiae*, and *Y*. *lipolytica.*

	***E. coli***		***S. cerevisiae***		***Y. lipolytica***	
	**Titer**	**References**	**Titer**	**References**	**Titer**	**References**
Fatty alcohols	1.8 g/L	Mehrer et al., 2018	6.0 g/L (Fed-batch)	d’Espaux et al., 2017	2.15 g/L (in a 3-L bioreactor)	Xu et al., 2016
FAEE	1.5 g/L (minimal medium)	Zhang F. et al., 2012	0.005 g/L	Runguphan and Keasling, 2014	1.18 g/L (containing 5 vol% ethanol)	Gao et al., 2018
FFAs	2.1 g/L (modified MOPS minimal medium)	Kim and Gonzalez, 2018	33.4 g/L (Fed-batch)	Yu et al., 2018	9.67 g/L (in a 3-L bioreactor)	Xu et al., 2016
Alkanes	0.426 g/L	Fatma et al., 2018	0.003 g/L (Delft minimal medium)	Zhu et al., 2017	58.7 mg/L (Fed-batch)	Bruder et al., 2019
γ-decalactone	ND	ND	Increase by 11%	Rong et al., 2017	8.37 g/L	Zhang et al., 2020
HFAs	275 mg/L	He et al., 2019	347 mg/L (Fed-batch)	Liu J. J. et al., 2019	7.9 g/L	Gatter et al., 2014
DCAs	ND	Wang F. et al., 2020	92.5 g/L (Fed-batch)	Lee et al., 2018	11 g/L	Gatter et al., 2014
Hexanal	ND	ND	ND	ND	increased by 84.1%	Aziz et al., 2016
CFA	ND	Guangqi et al., 2010	ND	Kochan et al., 2019	2319 mg/L	Czerwiec et al., 2019

*ND, not determine.*

## Conclusion and Future Perspectives

*Y. lipolytica* is a promising workhorse gaining great attention. Currently, the advance of metabolic engineering and synthetic biology enables *Y. lipolytica* to produce various value-added chemicals with different substrates and metabolic engineering strategies, including the design and construction of synthetic pathways, regulation of endogenous genes, and optimization of the fermentation process. However, several challenges remain in limiting the wide applications of *Y. lipolytica*.

When developing and optimizing *Y. lipolytica* for improving the production of value-added chemicals, the whole bioprocess, including the upstream of strain development and bioproducts production, the midstream of scale-up fermentation, and the downstream of recovery and purification, is needed to be considered first. Ko et al. (2020) showed that systems metabolic engineering, integrating systems biology, synthetic biology, and evolutionary engineering can enable microbial strains to efficiently produce chemicals. Therefore, systems metabolic engineering can be further applied to better manipulate the engineered *Y. lipolytica* to synthesize the desired bioproducts. Meanwhile, to optimize cell metabolism, such as reducing the negative effects of intermediate accumulation and metabolic perturbations, the dynamic metabolic engineering capable of tuning the cell growth and bioproducts formation is becoming a promising approach to better engineer the host strain (Xu, 2018). Moreover, due to the limits of dimorphic nature, cellular engineering and bioprocess engineering can be used to improve the yield of products at the industrial scale (Soong et al., 2019). Additionally, to reduce the cost of bioprocess, other low-value carbon sources, especially single-carbon substrates, will be utilized and converted to valuable fatty acid-based bioproducts by metabolic engineering *Y. lipolytica*. Conclusively, the application of *Y. lipolytica* for fatty acid-based chemicals production shows a great promise for researchers working in this field.

## Author Contributions

HL conceived the outline and revised the manuscript. YT finalized the topic of this review, and all authors wrote the manuscript. All authors read and approved the final manuscript for publication.

## Conflict of Interest

The authors declare that the research was conducted in the absence of any commercial or financial relationships that could be construed as a potential conflict of interest.
